# Early Intervention to Improve Hand Function in Hemiplegic Cerebral Palsy

**DOI:** 10.3389/fneur.2014.00281

**Published:** 2015-01-06

**Authors:** Anna Purna Basu, Janice Pearse, Susan Kelly, Vicki Wisher, Jill Kisler

**Affiliations:** ^1^Institute of Neuroscience, Newcastle University, Newcastle upon Tyne, UK; ^2^Department of Paediatric Neurology, Newcastle upon Tyne Hospitals NHS Foundation Trust, Newcastle upon Tyne, UK; ^3^Paediatric Physical Therapy, Newcastle upon Tyne Hospitals NHS Foundation Trust, Newcastle upon Tyne, UK

**Keywords:** cerebral palsy, early intervention, upper limb, elastic taping, thumb-in-palm deformity, hemiplegia, therapy, orthoses

## Abstract

Children with hemiplegic cerebral palsy often have marked hand involvement with excessive thumb adduction and flexion and limited active wrist extension from infancy. Post-lesional aberrant plasticity can lead to progressive abnormalities of the developing motor system. Disturbances of somatosensory and visual function and developmental disregard contribute to difficulties with hand use. Progressive soft tissue and bony changes may occur, leading to contractures, which further limit function in a vicious cycle. Early intervention might help to break this cycle, however, the precise nature and appropriateness of the intervention must be carefully considered. Traditional approaches to the hemiplegic upper limb include medications and botulinum toxin injections to manage abnormalities of tone, and surgical interventions. Therapist input, including provision of orthoses, remains a mainstay although many therapies have not been well evaluated. There has been a recent increase in interventions for the hemiplegic upper limb, mostly aimed outside the period of infancy. These include trials of constraint-induced movement therapy (CIMT) and bimanual therapy as well as the use of virtual reality and robot-assisted therapy. In future, non-invasive brain stimulation may be combined with therapy. Interventions under investigation in the infant age group include modified CIMT and action observation therapy. A further approach which may be suited to the infant with thumb-in-palm deformity, but which requires evaluation, is the use of elastic taping. Enhanced cutaneous feedback through mechanical stimulation to the skin provided by the tape during movement has been postulated to modulate ongoing muscle activity. If effective, this would represent a low-cost, safe, widely applicable early intervention.

## Introduction

Cerebral palsy (CP) is the commonest cause of neurological disability in children. The upper limbs are often affected, with significant wrist and hand involvement from an early age (1). Persisting from infancy, affected children may have abnormal hand postures such as thumb adduction and/or flexion with limited wrist extension, as well as more proximal abnormalities of upper limb tone, posture, and function, which also impact on hand use. The particular focus of this article is on the factors influencing hand structure and function in hemiplegic cerebral palsy (HCP), and the options for early intervention.

### Inter-relationship between body structure and function, activities and participation

The international classification of functioning, disability and health (ICF) (2) provides a framework, which describes the inter-relationship between body structure and function, activities and participation, as well as the influence of environmental and personal factors. In the case of hand function in HCP, we see how closely each factor impacts on the others, as discussed below. This can lead to a vicious cycle of deteriorating structure and function and maladaptive activity-dependent plasticity, but can also offer hope for early intervention approaches to break the cycle.

### Central nervous system deficits in HCP

Hemiplegic cerebral palsy affects around 1 in 1300 live births (3) and has a range of etiologies including neuronal migration abnormalities, periventricular leukomalacia, intracranial hemorrhage, and infarction. The common feature is disturbed cerebral control of motor function. A large component relates to corticospinal tract damage, as the corticospinal tract is the major descending tract controlling skilled, fractionated, voluntary hand movements (4). In addition, there is often extrapyramidal involvement (5); some patients have marked dystonia. As well as disruption of motor pathways, there are often sensory impairments including astereognosis (6–9), which impact detrimentally on hand function. These sensory impairments may reflect additional damage to ascending thalamocortical pathways (10, 11) and/or somatosensory cortical areas (9), as well as failure of sensorimotor integration (12). Disturbances of visual function, including but not limited to hemianopia, further contribute to difficulties with hand use (13). Deficits in motor planning and motor imagery as well as broader deficits in executive function are also seen (14–16). Finally, a minority of patients have significant learning disability, which can also impact adversely on the development of hand function (17, 18).

### Role of activity-dependent plasticity within the central nervous system

In addition to structural damage from the initial insult, progressive maladaptive changes occur within the central nervous system due to activity-dependent plasticity. This has been particularly studied within the corticospinal tract (19). Neurophysiological studies in humans indicate that in healthy term infants, corticospinal fibers from each hemisphere project to each side of the spinal cord (bilateral system), with gradual progression to a predominantly crossed projection within the first 2 years of life (20). If this system is perturbed by unilateral perinatal stroke, remaining corticospinal projections from the affected hemisphere may be gradually displaced over time by uncrossed projections from the undamaged hemisphere. This is in general associated with a worse functional outcome than the normal pattern of predominantly crossed corticospinal projections from each hemisphere (21). However, the relationship between corticospinal tract reorganization and functional outcome is complicated, and the nature, timing, and size of the lesion also play a role (22). A marked degree of corticospinal tract reorganization can occur (23).

Progressive maladaptive central nervous system processes in HCP are not limited to the corticospinal tract. Development of spinal cord segmental circuitry is influenced by corticospinal tract activity. If this is disrupted experimentally through transient motor cortex inhibition during early development, subsequent patterns of spinal cord circuitry including interneuron development are abnormal and immature (24–26). In addition, the M1 motor cortical map does not develop normally (27). However, processes such as early restraint of the unaffected limb combined with motor training (28), can reverse to an extent the changes in spinal cord circuitry, corticospinal tract projections, and M1 motor map abnormalities as well as improve motor outcome. Considering this in light of the ICF framework demonstrates that it is possible to alter structure and function either positively or negatively through modulation of activity.

### Effects on body structure and function

The implications of the above central nervous system changes on upper limb tone, posture, reflexes, and function in congenital hemiplegia are well-known but take time to emerge (17). In the first months of life the typical signs of a hemiplegia are not yet present, although qualitative abnormalities of movement may be detected (29). In addition, many healthy neonates and young infants will demonstrate features such as a thumb-in-palm posture, which disappear over the first few months of life (30). In contrast, the infant developing HCP will often have a closed hand posture and flexed and adducted thumb persisting beyond the first few months.

The development of asymmetrical hand function shows significant variation in infants with HCP; however, pronation of the forearm and thumb abnormalities is most frequently seen (31). As the infant develops reaching and grasping abilities on the unaffected side, parents start to notice a strong hand preference, with lack of use of the affected hand. The fingers are flexed, and the thumb is often adducted and flexed, resulting in the “thumb-in-palm” deformity (32). Dislocation may occur at the metacarpophalangeal joint, with hypermobility at the proximal interphalangeal joints causing swan-neck deformity, which impacts negatively on opposition and grasp (33). The thumb adduction also impacts on grasp, especially pincer and whole hand grasp (34). In a study of children age 4–14 years with CP, marked structural deformities affecting hand function were noted even in the youngest children, without significant age-related increases (1). Another study also reported structural deformities even at age 2 years (31).

More proximal upper limb deficits in hemiplegia also affect hand use. Increased muscle tone is noted predominantly in the upper limb flexor muscles with extensor weakness. Dynamically increased muscle tone is particularly clear in biceps brachii during physical activity such as walking, running, and even moving the dominant hand. Pectoralis major, the forearm flexors, and pronator teres exhibit hypertonia in some children, pulling the shoulder into flexion, adduction, and internal rotation, the forearm into pronation, and the wrist into flexion, often with ulnar deviation. Active forearm supination and wrist extension are limited (32). While some of the increased tone is due to spasticity or rigidity, there are also secondary biomechanical changes in the muscles leading to hypertonus, which is independent of recorded EMG activity, but which is temporarily reduced through muscle stretching (“plasticity”) (35, 36). Over time such increased tone can lead to the development of contractures. In addition, there is reduced limb growth on the affected side. Difficulty with selective muscle control and/or excessive co-contraction can discourage use of the hemiplegic limb which in turn compounds muscle weakness (37). Effective bimanual function is often limited (38), with increased reliance on one-handed strategies (39).

### Effect on activity and participation

Many daily activities require the hands to perform different movements at the same time in a coordinated way. Generally, the non-dominant hand assists by holding and stabilizing objects while the dominant hand performs more precise functional movements. The child with HCP has to battle with a number of hindrances in order to use the affected hand, and may make an active choice to use the “less-affected” hand alone. [Note that subtle deficits in function are present on the side contralateral to the hemiplegia – hence the term “less-affected hand” (40, 41)]. However, particularly for young children, the reasons for neglecting to use the affected hand are not always clear and the concept of “developmental disregard” has been coined (42). This has been described as the discrepancy between capacity and performance with the affected hand (43). Recent EEG studies indicate an increased cognitive load for movement preparation with the affected hand during a bimanual task (43). This could contribute to a preference for unimanual task performance. Compensatory strategies commonly observed include use of the teeth or stabilizing objects between the knees or against the body instead of using the affected hand. While children may be able to perform some “bimanual” tasks largely one-handed using such strategies, task completion time often remains prolonged (44). This can lead children to seek the assistance of others, or to avoid certain activities (45). Parents, teachers, and friends may step in too early to provide assistance. Even where the use of both hands is actively encouraged, very young children may not understand why this is important. However, as children get older they begin to recognize things they would like to be able to do more easily, particularly during the teenage years when personal independence becomes increasingly important. Personal and familial emotions and attitudes regarding the hemiplegia will influence for any individual the size of the gap between capacity and performance. These factors are not well described in the literature, though self-esteem and self-concept have been explored (46).

We have illustrated how structural abnormalities in HCP impact on hand function, activity, and participation, and how personal and environmental factors influence this. There is a need for safe, cost-effective, evidence-based early interventions for infants and young children at risk of developing hemiplegia (47). Intervening early could break the vicious spiral of declining structure and function and improve long-term functional outcome. Optimizing hand and wrist posture could encourage the child to experiment more with using the affected limb, drive use-dependent plasticity toward optimal solutions, and reduce the risk of secondary musculoskeletal problems. Consideration should also be made regarding how to enhance visual and sensory awareness of the affected arm and hand and promote its use in play and other activities.

## Established Medical and Surgical Interventions

Outside the period of infancy, medical and surgical interventions are often considered; these include medications, targeted botulinum toxin treatment, and eventually surgical approaches, with ongoing therapy input and orthoses as appropriate. Botulinum toxin can reduce spasticity in targeted muscles and improve range of movement at a particular joint, facilitating splinting and therapy. This is a time-limited intervention: effects wear off and repeated administration is required. There is concern regarding the long-term effects of repeated injection, resulting in weakness and muscle atrophy in a group of children for whom muscle weakness is already a problematic feature (48). Botulinum toxin is both unlicensed and rarely used in practice for children age <2 years although a recent review of off-label botulinum toxin treatment identified three randomized controlled trials including children in this age range (49). Only one of these was relevant to upper limb function (50), this included children age 22–58 months. The study, while small, indicated a benefit from repeated botulinum toxin injections in the upper limb over and above that of occupational therapy alone, with respect to spasticity and parental perception of performance.

Surgical approaches include tendon transfer, muscle lengthening, and arthrodesis. These well-established procedures offer permanent and fixed solutions: in the case of arthrodesis, the overall range of movement is reduced. Treatment goals include achieving a more functional hand position and improving appearance or hygiene of the arm and hand. Robust studies of long-term outcome are sparse, but there is evidence of benefit (51–53). This includes perceived long-term benefit in terms of function and cosmesis from the patient perspective (54, 55). A full review of medical and surgical interventions is beyond the scope of this article. Below, we focus on therapy and splinting approaches and how these might apply to the infant population.

## Therapy Approaches

A number of strategies have been developed aiming to improve hand function in children with established HCP, with varying evidence of benefit (56). Current therapy approaches fundamentally comprise repeated practice of desired movements (sometimes including shaping, i.e., breaking down the goal into incremental steps in line with progress), with the child as an active participant. Most activities consider the principles of motor learning and neuroplasticity and are adapted to the age and cognitive ability of the child (57). Approaches may be play-based, problem-solving, or goal centered, for example, around specific activities of daily living (58). Some approaches such as the use of videogames/virtual reality, or robot-assisted therapy (sometimes in combination with virtual reality games), represent alternative modes of delivery of upper limb therapy rather than radically new principles *per se* (59). In older children with hemiplegia, there are now studies combining non-invasive brain stimulation with occupational therapy approaches.

Some therapies are delivered in intensive bursts (e.g., 60–90 h over a few weeks), which differs from more conventional therapy approaches (57, 60, 61). Information regarding the optimal therapy “dose” is available for only a minority of interventions (62, 63). The practicalities of intensive therapy delivery must be considered in the light of other demands on the child, family, and therapist time as well as other resource pressures. Further information on the relative merits of intensive versus standard models of therapy will emerge in due course (64). A combined approach of infrequent intensive bursts of therapy, with lower-intensity “maintenance” therapy during interim periods, may prove optimal for enhancing and maintaining function.

Of key importance is the transfer of motor function gains from therapy to everyday life, without which there is little point in undertaking the therapy (65). This has been overtly assessed in the adult population in the context of constraint-induced movement therapy (CIMT – see below) (66). Incorporation of a “transfer package” with items such as a behavioral contract, home diary, task practice in the home setting, and ongoing telephone contact beyond the end of the therapy, greatly enhanced the improvements in the amount and quality of arm use compared with lab-based CIMT alone. There is indirect evidence from pediatric studies that similar principles apply (65).

### Challenges of early therapy approaches

Developing upper limb therapies suitable for infants is a challenge for many obvious reasons. Infants have short attention spans and little or no understanding of the need for therapy or its aims. This makes it difficult to develop approaches which will be tolerated. Furthermore, evaluating the effects of therapy is tricky in a group with ongoing developmental changes in whom the outcome without intervention can vary. While there is a range of assessments of general motor function in infancy (currently validated to varying degrees), there is only one *validated* assessment of upper limb function – the Mini Assisting Hand Assessment, which measures function of the affected arm during structured bimanual play in infants 8–18 months (67). A further assessment, the Hand Assessment of Infants (HAI) for measuring unimanual and bimanual function in infants age 3–12 months, is in the late stages of development (68, 69). The grasping and reaching assessment of Brisbane (“GRAB”) is being developed for infants from around 3 months (69) but this is also under development and not yet validated.

### Constraint-induced movement therapy and bimanual therapy

Two therapy approaches, which have received much recent attention, are CIMT, in which the use of the less-affected hand and arm is restricted to encourage use of the more-affected upper limb, and intensive bimanual therapy (62). There is evidence of significant benefit from both of these approaches (56). Bimanual therapy may require a greater intervention duration (62). Guidelines for future research have been drawn up in relation to remaining unknowns regarding constraint therapy, including long-term benefits, effect of repeated programs, effect on bimanual performance, and activities of daily living, optimal type of restraint, type and duration of training, environment where the training is delivered and which patient characteristics influence outcome (70). The long-term effects on function of the less-affected limb must also be monitored (41).

Considering the infant population specifically, there has, to date, been no definitive evaluation of CIMT. However, infants have been included as part of trials studying a broader age group (8 months to 8 years) (71). Similarly, in a non-randomized trial, constraint improved the use of the hemiplegic hand in bimanual play compared with standard therapy approaches in children age 18 months to 4 years (72). The approaches and challenges of CIMT in young children with hemiplegia have been addressed by Taub et al. (73). These challenges are now being addressed in infants at risk of HCP: Eliasson et al. (68) will compare baby constraint-induced movement therapy (Baby-CIMT) with baby massage. Baby-CIMT is described as a modified CIMT protocol, the restraint being a mitt or glove. The intervention will be for 30 min daily for 6 weeks in infants age 3–8 months and will focus on grasping and exploring objects in an enriched environment with close attention to carers’ and therapists’ behavior toward the infant, toy selection and position of the infant (68). With respect to the need for development of movement control of the “unaffected” hand and for bimanual motor integration in infancy, caution is needed when considering CIMT in infants (41, 70); however, this protocol represents a much reduced intensity of intervention compared with classical CIMT, which would typically involve 60 or 90 h of therapy within a few weeks (63).

### Action observation therapy

“Action observation therapy” is a method aimed at stimulating the mirror neuron system – a frontoparietal network of neurons activated both during action observation and action performance. Mirror neurons were first identified by Rizzolatti in animal studies and were so named because they fired when the animal either performed a motor task or observed the same task being performed (74). There is indirect evidence for the existence of a human mirror neuron system (75). Therapies incorporating repeated action observation and imitation showed promise in adults with stroke (76, 77).

Several small studies in children with HCP, generally adopting a bimanual approach in contrast to CIMT, also suggested that a benefit from action observation therapy (78–80). One larger study from our own group (ISRCTN65947097) in children with HCP age 3–10 years has also recently completed. There is to our knowledge only one study exploring the possible benefits of action observation therapy on upper limb function in infants at risk of developing HCP, as well as in healthy control infants (69). The intervention is targeted between the 9th and 13th weeks of life, during which parents will repeatedly demonstrate grasping actions on specific toys. This will be compared with presentations of the toys without demonstrating appropriate grasping actions. The effects on quantity and quality of reaching and grasping, as well as neurophysiological measures, will be assessed. Thus, at present, the role of action observation therapy, though intuitively appealing as a natural way of learning through watching and copying, remains to be established.

## Hand Splints/Orthoses

A splint has been broadly defined as “a brace, orthosis, cast, tape, or any external device applied to one or more joints” (81). Hand splints may be divided into “non-functional” splints aimed at preventing contracture by providing prolonged stretch, and “functional” splints aimed at improving motor task performance by supporting joints in biomechanically optimal positions (82). Although upper limb orthoses are frequently prescribed by therapists for children with hemiplegia, compliance overall is under 50% (83), with ineffectiveness and refusal to wear the orthosis being reasons for abandonment. Pain, discomfort, and reduction in function have been reported in the adult stroke literature (84). Additionally, by covering part of the palmar surface, thumb, and volar splints reduce palmar sensory feedback, which is vital for sensorimotor integration. All orthoses have the advantage that they can be worn for short periods of time rather than continuously, reducing the risk of disuse atrophy compared with approaches such as casting.

“Non-functional” orthoses offer a benign, temporary solution to correcting posture but are relatively inflexible. This reduces function and with excessive use may lead to muscle atrophy and compensatory overuse of other muscle groups (85). Therefore, non-functional orthoses are often used either at night or for short periods of time to achieve a particular goal, for example, increased muscle length. However, the evidence for this approach (86) or indeed for the benefit of non-functional orthoses (82) is limited. A recent systematic review found five randomized studies of non-functional upper limb splints (82), showing a small immediate benefit for hand function, which was not maintained. The review identified only one study of upper limb functional splinting, in which a Lycra splint from the wrist to the axilla had an additive benefit on goal attainment scores compared with goal-directed training alone (87).

An early description of a functional thumb splint was provided by Currie and Mendiola (88). The splint was assessed in just five children with HCP, aged 20–26 months. In each child this immediately improved thumb position, grasp type, and spontaneous bimanual exploration. More recently, Louwers et al. (89) studied the immediate effects of wearing a thumb and wrist brace on bimanual hand function in 25 children age 4–11 years with HCP. Performance on the Assisting Hand Assessment (which measures the spontaneous use of the paretic hand to assist in tasks requiring bimanual function) (90) increased by an average of 3.2 raw scores simply by wearing the brace, and 52% of children improved by at least 4 points. No attempt to look at longer-term use, acceptability, or tolerability was made in either of these studies. Ten Berge et al. (91) studied the effects of a 2-month intervention with a neoprene thumb opponens splint on hand function in seven children age 2–7 years with hemiplegia. The splint was prescribed for at least 4 h/day. Compliance was good but goal attainment scores increased above baseline variability in only three patients.

Splints can be particularly challenging for use in young children, who may simply remove them. It is difficult to create well-fitting splints for very small hands and wrists given the nature and thickness of the materials used. Until a clearer evidence base emerges, decisions regarding splinting are fraught with uncertainty (82). In the light of the inadequate evidence base (82), Jackman et al. propose to undertake a study comparing the effects of functional hand splints alone, with task specific training alone, and with both approaches combined, in children age 4–15 years (81).

## Elastic Taping

An approach increasingly being used for the upper limb in neurorehabilitation settings (92) but which, to date, has not been adequately evaluated, is the use of elastic taping. This differs from the more rigid forms of “athletic taping” originally developed for sports injuries. Hypoallergenic, waterproof versions of elastic tape are available, as are a range of colors, which can help in attracting the infant’s visual attention (though natural, skin tone colors are sometimes more helpful for infants who tend to remove the tape). Specific manuals and training courses are in existence and various methods of application have been described, including a “thumb extension assist” approach for the thumb-in-palm deformity (93). With caution, parents could be trained in application by an appropriately qualified therapist. This is an important consideration because application needs to be repeated frequently (around twice a week) (94).

Many claims have been made regarding the potential benefits of functional elastic taping and these are counterbalanced by both a healthy skepticism and lack of clear evidence of efficacy (95). One of the more plausible stated mechanisms of action relevant to HCP is through enhancement of cutaneous sensory feedback via stretch applied to the skin during movement. For example, taping for the thumb-in-palm deformity includes a longitudinal strip under tension on the dorsum of the thumb, which could lead to increased firing of cutaneous afferents (mechanoreceptors) on the underlying skin during thumb flexion. This could lead to enhanced proprioceptive feedback (96, 97). In fact, skin strain patterns in the hand provide kinesthetic information taking precedence over that from muscle spindle and articular afferents in some situations (98); this kinesthetic role may have been underestimated in the past (99). Complex interactions at spinal cord level lead to integration of signals from the various proprioceptive afferents (100), which can then affect muscle spindle sensitivity through modulation of gamma motor neuron firing (101–103), and ultimately perhaps alter the balance of muscle activity to strengthen thumb extensors over time (104).

This provides the theory; however, the evidence in practice for kinesthetic benefits is lagging. While rigid, athletic tape can stabilize the ankle joint and improve proprioception (105, 106), studies of elastic taping have produced mixed results (107, 108). Additionally, studies in CP as well as in control populations at different ages are essential. This is because cutaneous reflexes show age-dependent changes and are altered in CP. Short-latency cutaneous reflexes are known to be largest in the first year of life, and are also exaggerated in the presence of an upper motor neuron lesion (109). In infants and in CP, stimulation of the digits can produce short-latency excitation of both the forearm flexor and extensor muscles, leading to co-contraction. In the second year of life, long-latency cutaneous reflexes emerge, taking a supraspinal route to the cortex with the efferent volley through the corticospinal tract (109), thus, the long-latency cutaneous reflex is affected in patients with an upper motor neuron lesion. The degree to which patients with CP would demonstrate a kinesthetic advantage from taping is unclear.

Studies in children with CP have tended to focus on practical outcomes such as gross motor development. For example, Iosa et al. (110) studied the effect of taping at the ankle joint in eight children with unilateral spastic CP. Taping was undertaken for 6 months, and led to a greater increment in Gross Motor Function Measure (GMFM) scores (assessed with the tape off) than expected within that timescale, as well as improved gait. The mechanisms behind this were not explored. Interestingly, the only patient who did not improve had marked dyspraxia with sensory integration dysfunction. Similarly, Simsek et al. (111) applied tape for a 12-week period to the paraspinal muscles, aiming to improve sitting posture in children with CP and GMFCS III–V. Fifteen children were randomized to the intervention and 15 were controls. Both groups also received physiotherapy. Sitting Assessment Scale scores (SAS) (112) did not differ at baseline but, were higher in the intervention group (with the tape removed) at 12 weeks. GMFM scores did not improve. Footer also found no benefit of paraspinal muscle taping on GMFM scores over a 12-week period (113).

Elastic taping has been used to improve upper extremity function in adults following stroke, with anecdotal benefit (92). It has also been used for the upper limb both proximally and distally in an acute rehabilitation setting in children with acquired brain injury (114). Children were tested with the Melbourne Assessment of Unilateral Upper Limb Function (MUUL) (115) first with the tape off then with tape applied according to perceived need. Significant improvements were seen both immediately and after 3 days. The range of different taping regimens and the sample heterogeneity pose some problems in interpretation. Mazzone et al. (116) also studied upper limb taping in 16 children with HCP, with a mean age of 3 years. They underwent a 17-month rehabilitation program with tape applied in the first and last 5 months and a 7-month washout period in the middle. Taping included the thumb and spiraled up the arm to the middle third of the humerus. The results were suggestive of improvement only in the taped periods, with the caveats that dropout rate was high (50%) and the assessment used (MUUL) was not suitable for children aged below 5 years.

We are unaware of any other published literature on the effectiveness of elastic tape in improving hand and wrist position and function in infants with CP. As it is increasingly being used in practice, a formal evaluation would be timely to determine if children with thumb-in-palm deformity benefit from this intervention, and if so, how.

## Conclusion

Early intervention for the upper limb in hemiplegia remains challenging, though progress is being made. We have touched earlier on the difficulties of outcome assessment in the youngest infants and children, which make evaluation of interventions very difficult. The diversity of the population under study, in terms of lesion type, differences in post-lesional reorganization, and the degree to which other factors such as vision, sensation, and cognitive ability impact on hand function, must also be considered. Studies tend to be small and are dominated by short-term outcomes (with the aim of avoiding confounders due to developmental trajectories), whereas the long-term outcome may be the more important consideration. Therapy approaches including CIMT and action observation therapy are now being actively explored in the youngest children and infants. Functional elastic taping has some potential merits in this age group but requires further investigation with properly controlled studies. These should include assessments which could shed light on the underlying mode of action.

## Author Contributions

Anna Purna Basu drafted the manuscript, to which Janice Pearse, Susan Kelly, Vicki Wisher, and Jill Kisler contributed substantially. All authors have seen, agree to, and are prepared to be accountable for the final version of the manuscript.

## Conflict of Interest Statement

The Guest Associate Editor Gavin John Clowry declares that, despite having previously collaborated with Anna Purna Basu and Janice Pearse and being affiliated to the same institution as author Jill Kisler, the review process was handled objectively and no conflict of interest exists. The authors received funding from the British Academy of Childhood Disability/Royal College of Pediatrics and Child Health (Paul Polani award) to undertake a pilot study of functional taping to improve thumb and wrist posture and function in children with cerebral palsy.
